# Total knee arthroplasty in ochronosis: a case of intraoperative patellar tendon rupture and review of literature

**DOI:** 10.1007/s11845-025-04268-w

**Published:** 2026-01-15

**Authors:** Kiran K. V. Acharya, Iknoor Singh Mann, Sachin Kumar

**Affiliations:** Department of Orthopaedics, Kasturba Medical College, Manipal, Manipal Academy of Higher Education, Manipal, Karnataka 576104 India

**Keywords:** Total knee arthroplasty, Ochronosis, Patellar tendon rupture

## Abstract

**Background:**

Ochronosis is a rare metabolic disorder characterized by the deposition of homogentisic acid in connective tissues, leading to fragility and degeneration. Total knee arthroplasty (TKA) in ochronotic arthropathy is challenging due to compromised bone and soft tissue quality. Patellar tendon rupture during TKA in this context is an uncommon but serious complication.

**Case Presentation:**

A 64-year-old male with severe degenerative knee arthropathy underwent left TKA. Intraoperatively, blackish discoloration of cartilage and tendons consistent with ochronosis was noted. During patellar eversion, a partial-thickness rupture of the friable patellar tendon occurred. The rupture was repaired. Postoperative recovery included early mobilization with a range-of-motion brace. At six months, the patient regained a knee range of motion from 0 to 120 degrees. Histopathology and biochemical testing confirmed ochronotic arthropathy.

**Conclusion:**

This case highlights the risk of patellar tendon rupture during TKA in ochronotic patients due to altered tendon integrity. Preoperative recognition and cautious handling of tendons are crucial. Early repair and rehabilitation can lead to favorable functional outcomes. Preoperative tendon evaluation by ultrasonography may help anticipate such complications.

## Introduction

Alkaptonuria disorder is an extremely rare disease characterized by black pigmentation of various tissues. It is caused by a deficiency of the enzyme homogentisic acid oxidase, which causes excess homogentisic acid, a catabolism intermediate in tyrosine metabolism, to accumulate in cells and body fluids, leading to dysfunction in the bone, cartilage and other connective tissues [1]. These tissues are characterised by a dark deposit of homogeneous acid due to a lack of homogeneous acid oxidase enzyme present in the liver and kidney [2, 3]. Blackening of the joint may be due to endogenous ochronosis(deficiency of homogentisic acid oxidase) or, rarely, exogenous ochronosis (due to accumulation of hydroquinone, resorcinol, phenol, mercury or picric acid) [1]. It happens in about 1 in 125,000 to 1 in 1 million people worldwide [4]. Ochronotic arthropathy is a debilitating disease affecting mainly the large joints. As with other metabolic diseases involving the musculoskeletal system, care should be taken to ensure the quality of the affected bones, tendons and ligaments and thus the stability and survival of the prosthesis. Ochronotic arthropathy is characterized by premature degenerative changes in the joints. We present a case of a patient with ochronotic arthropathy who underwent total knee replacement on the left side, during which the patellar tendon was found to be fragile. A partial tear in the tendon was identified and successfully repaired during the surgery.

## Case report

**Fig. 1:**
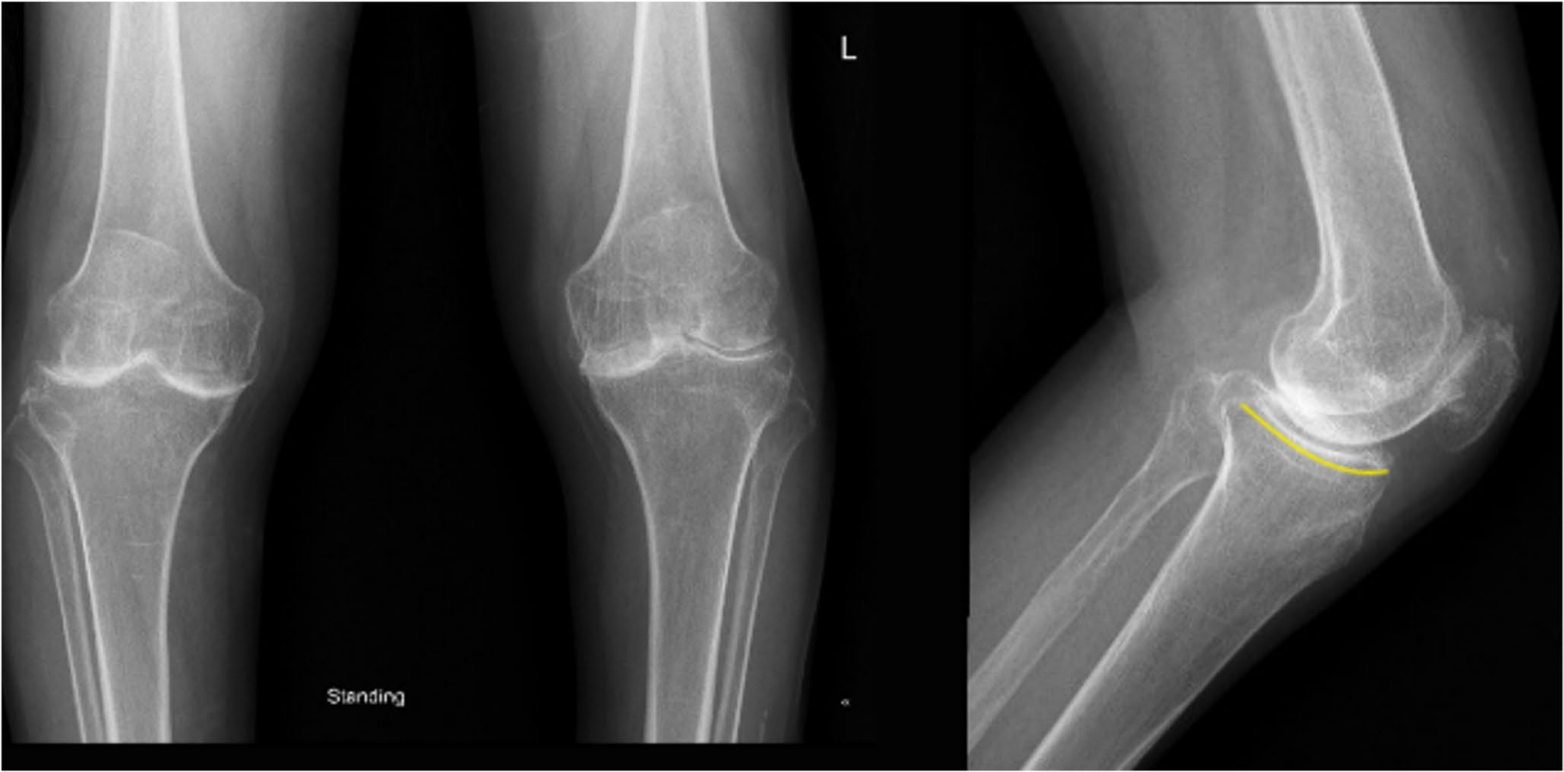
Bilateral knees AP radiograph in standing position shows severe degenerative change in his left knee with osteophytes and symmetrical reduced joint space with dished out proximal tibia articular surface (highighted line) and sclerosis noted over femoral condyles in AP view over bilateral knees

**Fig. 2:**
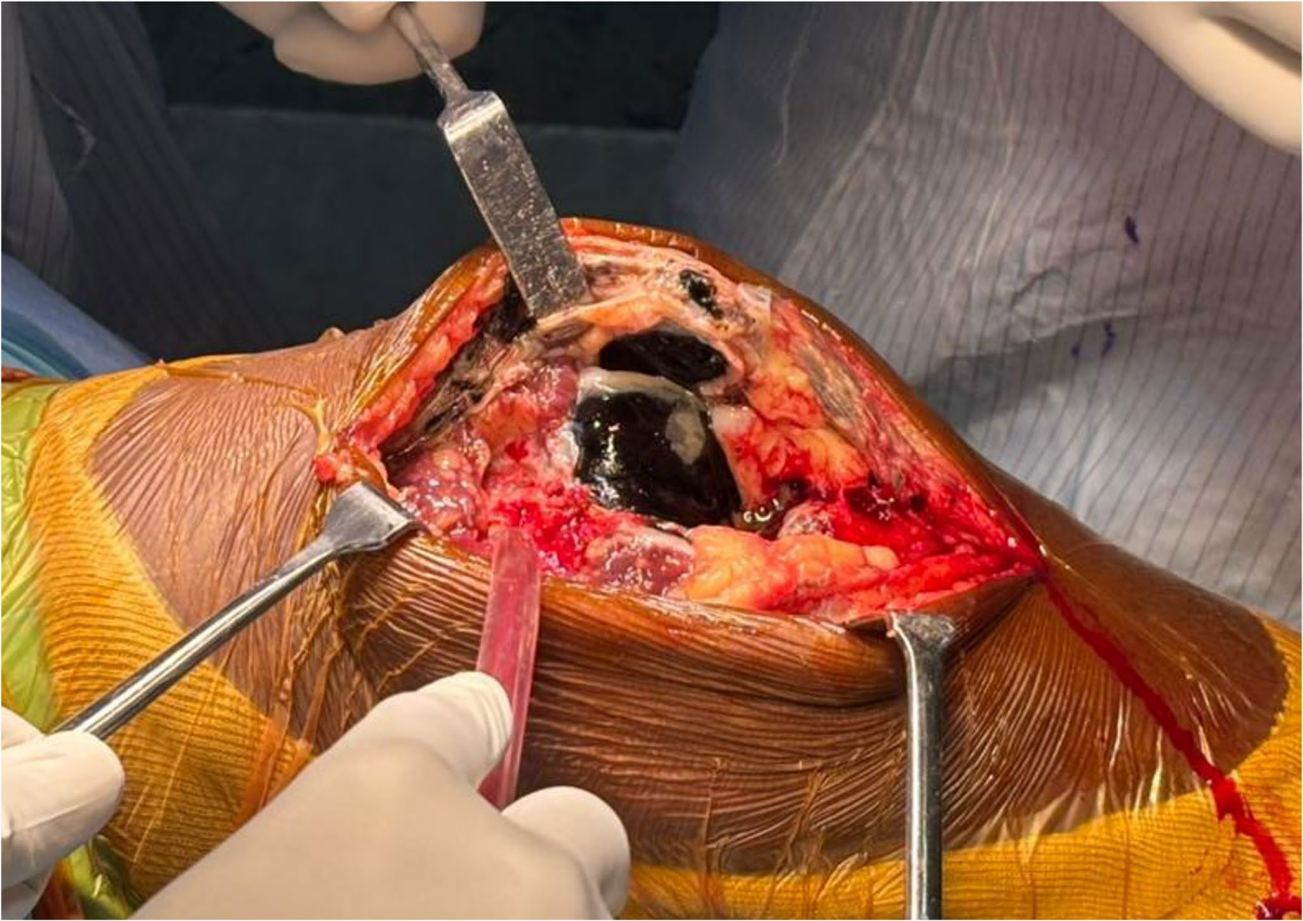
Intra op pictures showing ochronosis of femoral condyles of left knee

**Fig. 3:**
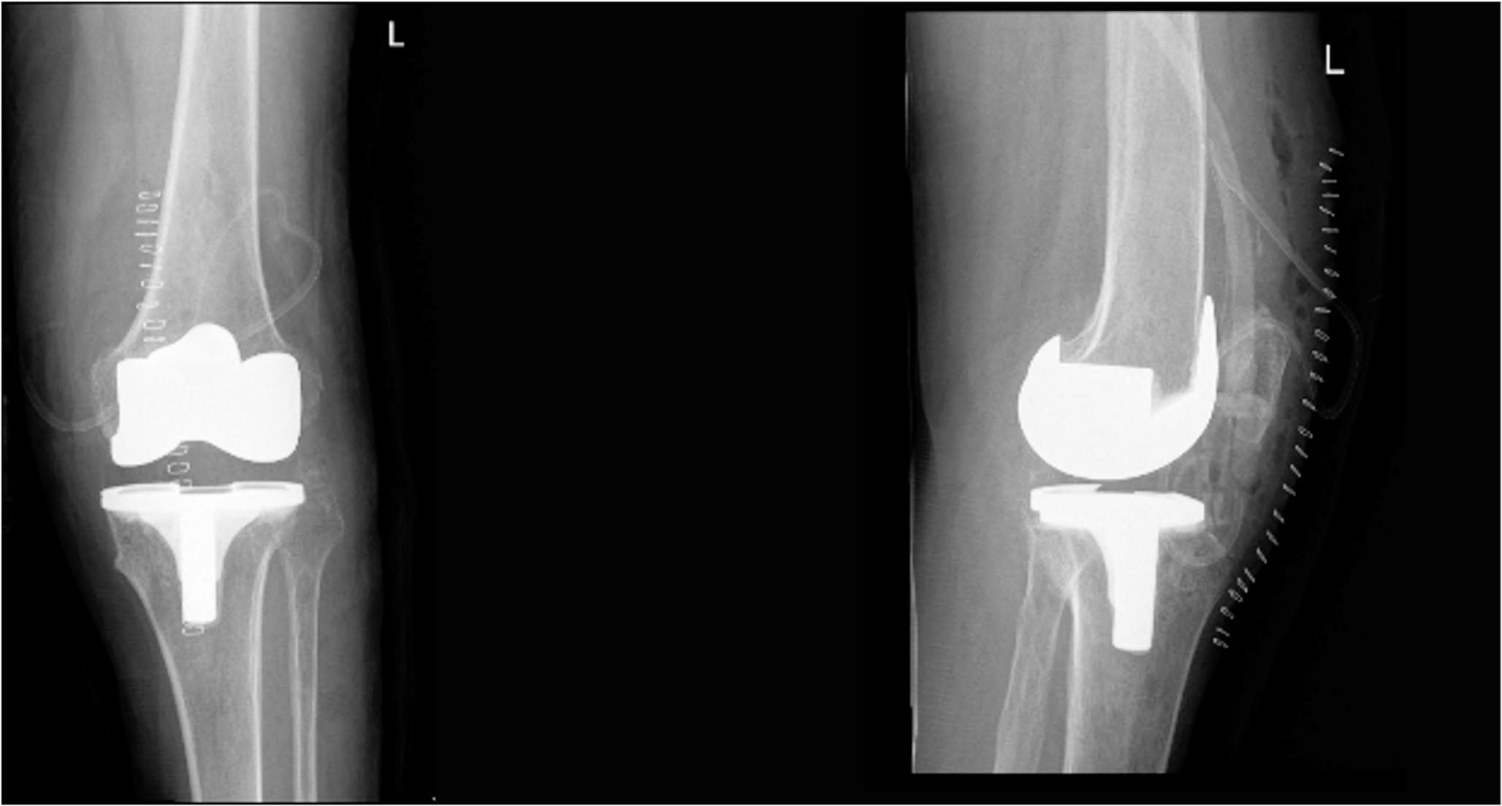
Post operative AP and lateral radiograghs of left knee showing orthopedic implant insitu and skin staples

A 64-year-old man presented to our hospital with history of bilateral knee pain left more than right. He had progressive limitations in daily activity for the past 6 years. There was no history of any trauma. Examination of the patient showed bilateral medial joint line tenderness and terminally restricted range of movements. Radiographs showed severe degenerative changes in his left knee with osteophytes and dished-out proximal tibial articular surface (Fig. 1). A diagnosis of primary osteoarthritis was made and left total knee arthroplasty was planned. A cemented posterior stabilized total knee arthroplasty was performed on the patient’s left knee with his informed consent. A standard midline incision was used. On exposure, the quadriceps tendon, patellar tendon, femur, tibia and patellar articular cartilage showed blackish discoloration (Fig. 2). The possibility of ochronosis was considered, intra-op culture sensitivity and tissue biopsy from bone cuts and synovium was sent. During everting the patella, as patella tendon was friable due to abnormal pigment deposition, there was partial thickness patellar tendon rupture from the tibial tuberosity. After implantation thorough wash was given, the ruptured tendon was repaired with No. 2 fiber wire, intermittent suturing done for partially torn patellar tendon directly to the bone, followed by augmentation using the Bunnell stitch through the tendon and a drill hole in the tibial tubercle. Patellar tracking was checked and wound was closed in layers over suction drain. Figure 3 shows the postoperative X-ray of the case.

**Fig. 4:**
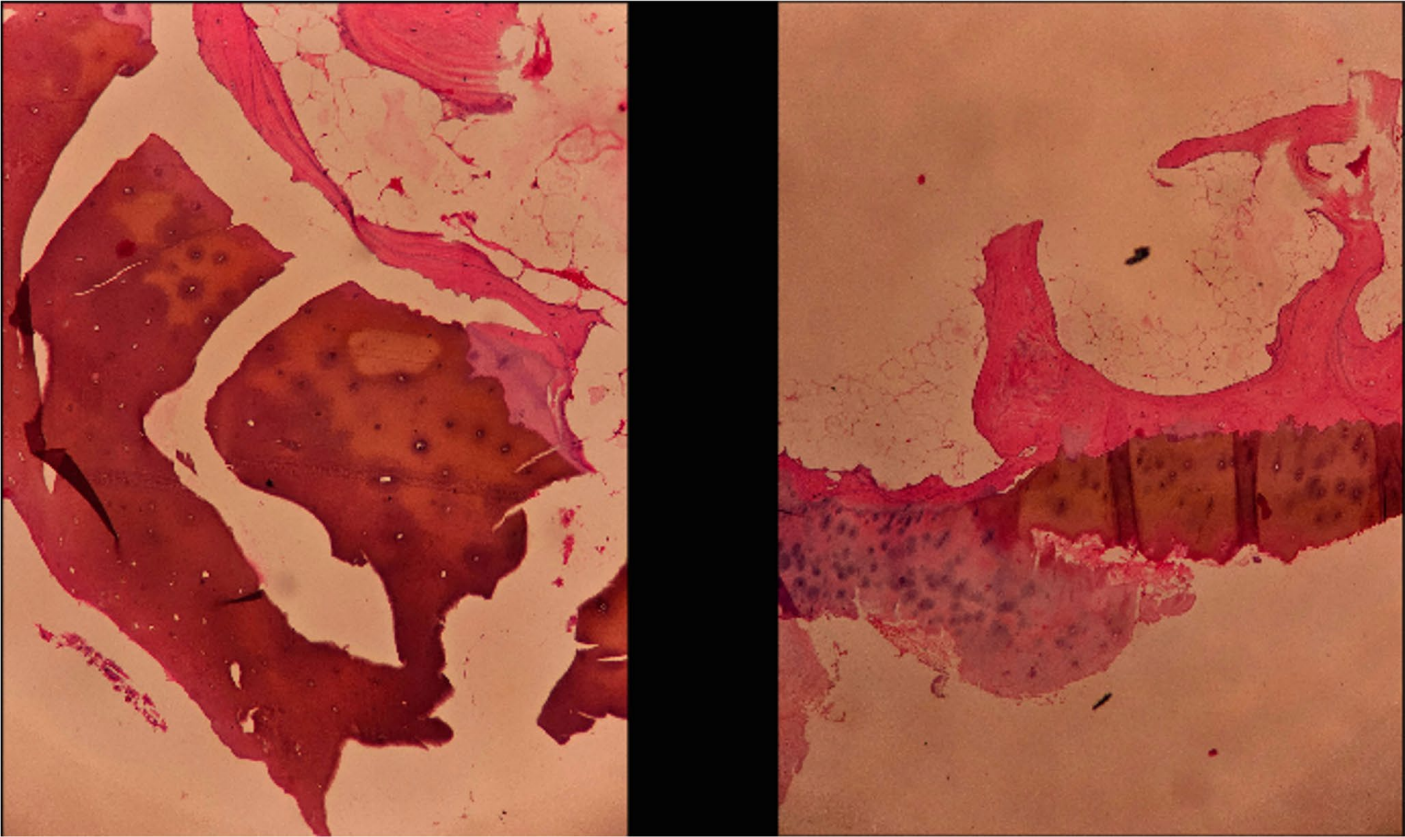
Cartilge pigmentation suggestive of Ochronosis

Post-operative day 1 patient was made to mobilize partial weight bearing with walker support and knee was protected with knee ROM brace. Knee was mobilized 0–90 degree with support of ROM brace for a period of 3weeks. Intra-op cultures were negative. A retrospective provisional diagnosis of ochronotic arthritis was made based on biopsy report (Fig. 4) and positive urine homogentisic acid postoperatively. Cervical spine X-ray also showed degenerative changes with significant osteophytes.

## Discussion

**Fig. 5:**
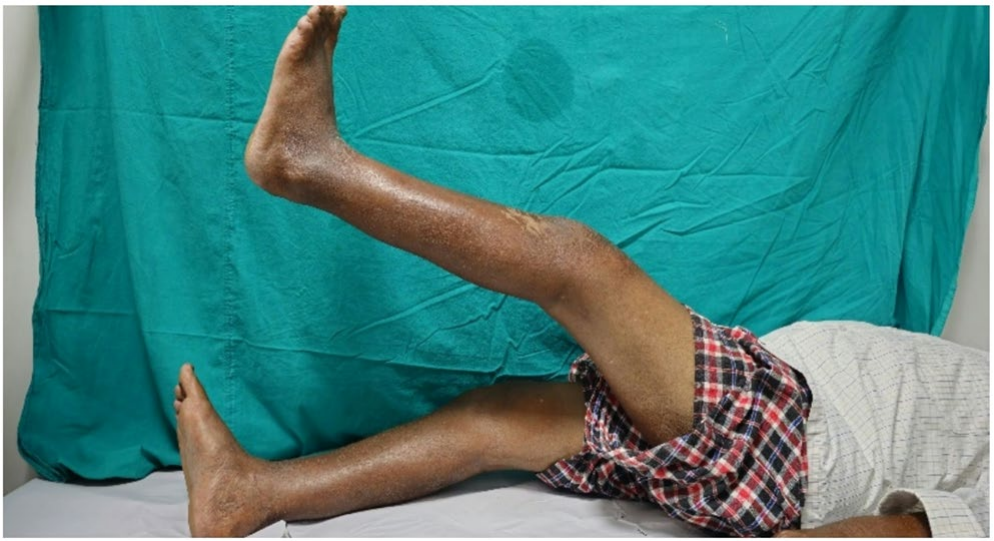
Knee in extension

**Fig. 6:**
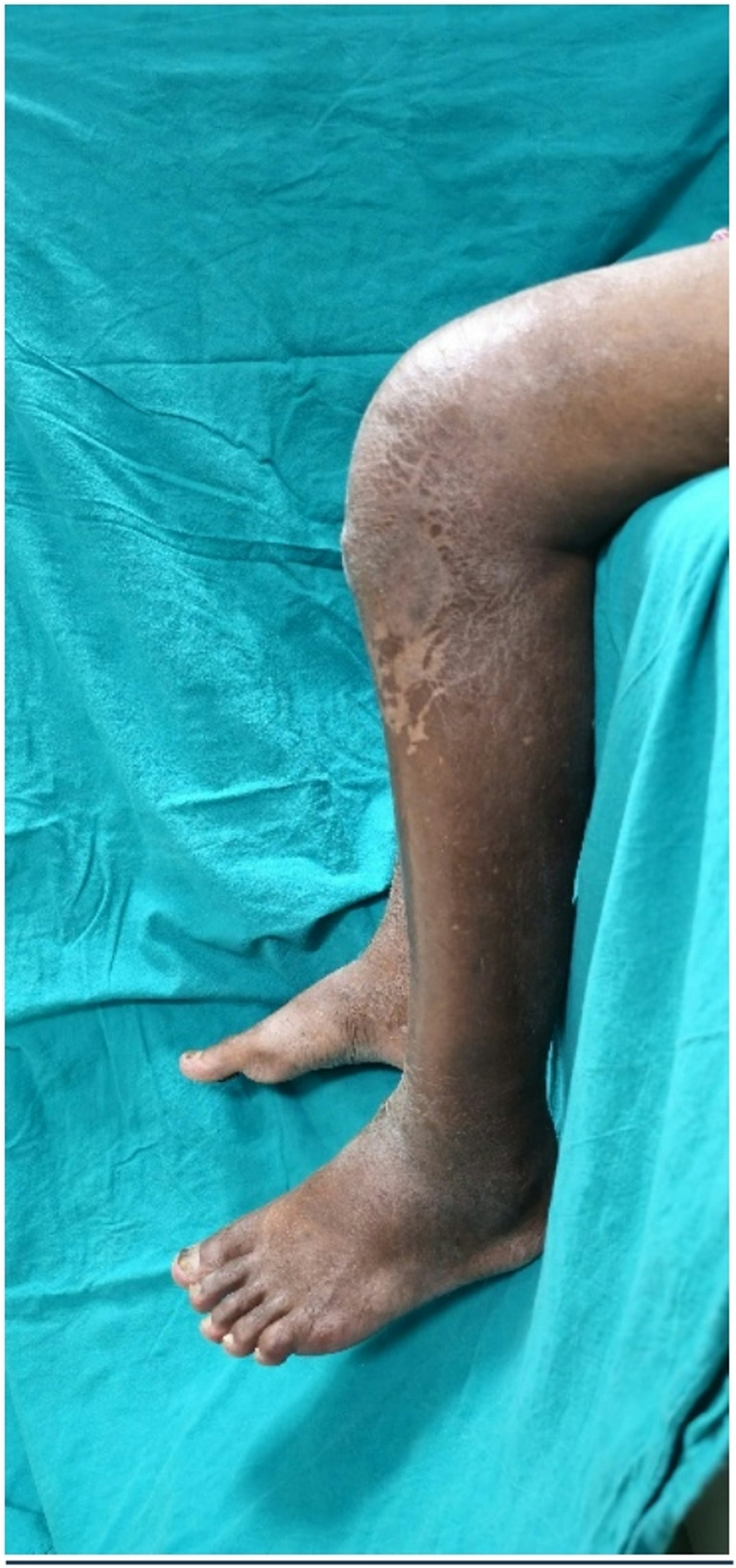
Left knee ROM after suture removal

Alkaptonuria causes bluish-black discoloration and weakness of some connective tissues. Ochronosis is the result of a long-term deposition of homogeneous acid in the cartilage, which increases the elasticity, allowing for cracking and premature degeneration. The flakes then attach to the synovium, causing thickening, fibrosis and chondromatosis [5]. A rapidly progressive arthritis then ensues by the 4th decade of life. This is an uncommon cause of progressive arthropathy and is diagnosed in most cases only upon intraoperative examination. The most common musculo-skeletal manifestations of osteoarthritis are the spine (intervertebral disc calcification and intervertebral space narrowing) and large peripheral joints (hip, knee and shoulder). Kumar et al. [6] reported a case of spontaneous rupture of the patellar tendon at the proximal part and 3 spontaneous ruptures of the Achilles tendon in 2 patients. The authors noted pigmentation at the rupture sites and fibrillation and degeneration of tendons and suggested that deposition of homogentisic acid inhibits collagen cross-linking in these tendons, which are predominantly composed of type 1 collagen. This leads to reduction of the structural integrity of the collagen, thus increasing the chances of rupture. Ando et al. examined a case of bilateral ruptured Achilles tendon and found that the ruptures were not lined with normal collagen [7]. Jebaraj et al. noted loss of fibrillary pattern in the tendon, increased thickness and small foci of calcification during ultrasonography in a case of Achilles tendon rupture [8]. Treatment for ochronotic degenerative arthropathy could be started as for degenerative osteoarthritis, with therapies such as analgesic drugs, nonsteroidal anti-inflammatory drugs (NSAIDs), rest, physiotherapy, and local injections. In case of severe arthropathy, where conservative treatment is not effective and the only alternative is replacement of the bone [1], care should be taken to take into account bone weakness and changes in tendons and ligaments, with a high risk of intraoperative complications affecting the stability and survival of the prosthesis. Patellar tendon rupture during TKR is a major complication that may occur when the knee is tight or has scarring due to previous surgery and when flexed with the dislocated patella. Various treatment options for these ruptures have been described, including fixation with suture or staples, autologous tissue augmentation with hamstring tendon, turndown of the quadriceps tendon or medial gastrocnemius flap or reconstruction with Achilles tendon allograft. In the present case, we believe that the pre-existing tendon degeneration could be responsible for rupture of patella tendon. We used the direct repair method and started protected knee mobilization with knee ROM brace. Knee mobilization was started early because these kinds of joint are more prone to develop stiffness. After suture removal patient had 0°−90° with 10° extensor lag (Figs. 5 and 6). 6 months post operatively patient is mobilizing well without support and 0°−120° range of motion with no extensor lag.

**Table:** 

Phase	Precautions and learning points
Preoperative	Maintain high suspicion via history (e.g., ear lobe discoloration, dark urine) and imaging; obtain cardiac clearance for possible valve calcification; use ultrasonography for tendon assessment [9].
Intraoperative	Anticipate dark pigmentation and friable tendons/cartilage; evert patella gently or excise osteophytes early to prevent rupture; use tourniquet, tranexamic acid, and cautious synovectomy to control bleeding; prefer cemented or posterior-stabilized implants for soft bones/ligaments [10]. Cemented implants are often preferred in ochronosis due to soft subchondral bone observed intraoperatively, which may compromise initial fixation stability in uncemented designs; multiple drill holes in cut surfaces enhance cement interdigitation [11].
Repair/Complications	Prepare for patellar/quadriceps tendon rupture with sutures, wire, or augmentation; ensure patellar tracking and have backup implants ready; address juxta-articular osteopenia/sclerosis promptly [9].
Postoperative	Initiate early protected mobilization with ROM brace; monitor blood loss and delay drain removal if needed; cautious quadriceps exercises due to stiff tendons [12].
Prognosis	Arthroplasty in ochronotic arthropathy shows comparable mid- to long-term success; incidental diagnoses do not worsen outcomes if managed [11].

## Conclusions

Our case report demonstrates that total knee arthroplasty has an excellent outcome in patients with severe degenerative arthropathy, secondary to ochronosis. Considering the high frequency of TKRs in ochronotic arthritis, the risk of intraoperative patellar tendon ruptures due to degeneration of tendons can be significant. Therefore, preoperative diagnosis is essential so that greater care can be taken in handling the tendon during TKR and repair can be undertaken in case of any rupture. A preoperative ultrasonography of the patellar and Achilles tendons may also help anticipate such complications in advance.

## Declarations

### Disclose generative AI use

Not used.
